# Forna (force-directed RNA): Simple and effective online RNA secondary structure diagrams

**DOI:** 10.1093/bioinformatics/btv372

**Published:** 2015-06-22

**Authors:** Peter Kerpedjiev, Stefan Hammer, Ivo L. Hofacker

**Affiliations:** ^1^Institute for Theoretical Chemistry, University of Vienna, Währinger Straße 17/3, A-1090 Vienna, Austria and; ^2^Research Group Bioinformatics and Computational Biology, University of Vienna, Währinger Straße 29, A-1090 Vienna, Austria

## Abstract

**Motivation:** The secondary structure of RNA is integral to the variety of functions it carries out in the cell and its depiction allows researchers to develop hypotheses about which nucleotides and base pairs are functionally relevant. Current approaches to visualizing secondary structure provide an adequate platform for the conversion of static text-based representations to 2D images, but are limited in their offer of interactivity as well as their ability to display larger structures, multiple structures and pseudoknotted structures.

**Results:** In this article, we present forna, a web-based tool for displaying RNA secondary structure which allows users to easily convert sequences and secondary structures to clean, concise and customizable visualizations. It supports, among other features, the simultaneous visualization of multiple structures, the display of pseudoknotted structures, the interactive editing of the displayed structures, and the automatic generation of secondary structure diagrams from PDB files. It requires no software installation apart from a modern web browser.

**Availability and implementation:** The web interface of forna is available at http://rna.tbi.univie.ac.at/forna while the source code is available on github at www.github.com/pkerpedjiev/forna.

**Contact:** pkerp@tbi.univie.ac.at

**Supplementary information:** Supplementary data are available at *Bioinformatics* online.

## 1 Introduction

The use of secondary structure diagrams is ubiquitous within the field of RNA biology. They convey not only which nucleotides are paired, but also and perhaps more importantly, which are unpaired. The contents and positions of sub-structures such as hairpin loops, interior loops, multiloop junctions and external loops are immediately evident. Such information is of great value to researchers seeking to identify putative mutations to perform when seeking to isolate the structural basis of a biological effect, to find protein binding, and to provide a context for observed behavior. It is used as both an exploratory as well as a communicative tool. Researchers examine secondary structure diagrams to gain insights about potential functions and mechanisms as well as to describe and disseminate them.

Although there are a number of available tools (Byun and Han, 2009; Darty *et* *al.*, 2009; Hecker *et al.*, 2013; Wiese *et al.*, 2005) for visualizing the secondary structure of RNA molecules, with the exception of PseudoViewer, none are available online without java and none offer the flexibility in exploring, arranging and manipulating the structure that forna does (Table 1 for an enumerated comparison of features).

**Table 1.** Comparison of the features of existing RNA visualization tools (where PV = PseudoViewer)

	forna	VARNA	PV	jVizRNA	RNAfdl
Editing	✓	✓			✓
Pseudoknots	✓	✓	✓	✓	
PDB files	✓				
Struct. prediction	✓				
Probing data	✓	✓			
Custom coloring	✓	✓			✓
Color schemes	✓	✓			
RNA-RNA pairs	✓	✓	✓		
Circular RNA	✓				
Annotations		✓			
Circular layout		✓		✓	✓

forna provides at least three convenient features not found in other programs.

## 2 Approach

**Fig. 1.**
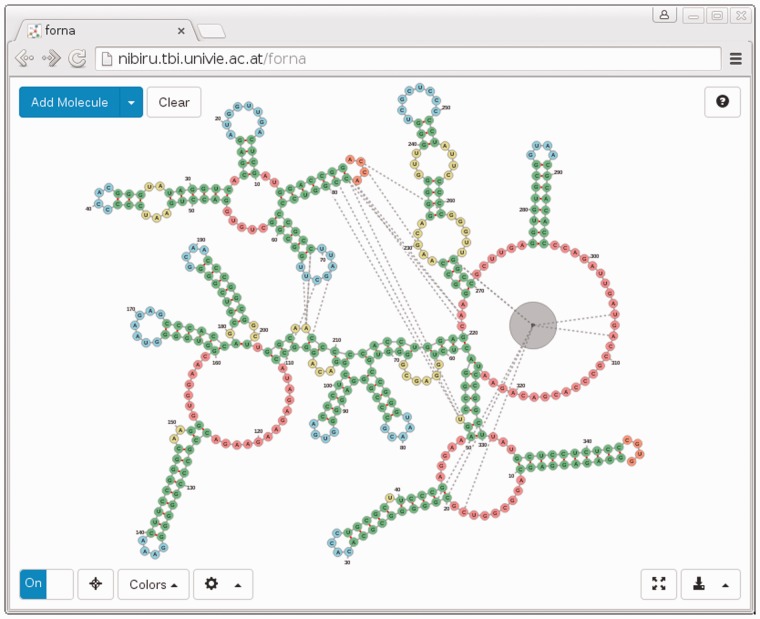
Screenshot of forna web app displaying the ‘Bacterial Ribonuclease P Holoenzyme in Complex with tRNA’ (PDB ID: 3Q1Q). Immediately evident are the regions of the tRNA which are in contact with the ribonuclease, namely the 5′ and 3′ end nucleotides, as well as the TΨC loop. An RNA-binding protein is shown as a gray node in the lower-right hand region of the diagram

Our tool, called forna (for force-directed rna), consists of a web interface and a server which allows users to input RNA secondary structures as dot-bracket strings, and displays it as a force-directed graph (Screenshot in Fig. 1). In a manner previously demonstrated by jViz.RNA (Wiese *et* *al.*, 2005) the user can then position each of the nucleotides and stems by dragging them. Each of the nucleotides is represented as a node, whereas backbone and base-pair bonds are considered links. Connections are treated as springs and a force is calculated to keep them a fixed distance from each other. Hidden helper nodes and extra links help to maintain the familiar RNA secondary structure layout. The initial position of each node (nucleotide) is calculated using the NAView algorithm (Bruccoleri and Heinrich, 1988), but is subsequently optimized by the force-directed layout algorithm. This can (especially for larger molecules) lead to artifacts such as twisted helices and nested loops, but these are easily rectified by dragging the affected nodes to their correct positions.

### 2.1 Input/output

Users can enter structures in dot-bracket format (Supplementary Material Sections S1.3, S1.6 and S1.7). When done, the diagram can be saved as either a vector (SVG) or raster (PNG) graphic. If one wishes to edit the structure again in the future, it can be saved and reloaded in forna using the JSON format.

### 2.2 Dragging to position elements

The layout can be rearranged by selecting and dragging single or multiple nodes. The virtual forces then pull the structure toward an RNA-like layout with nearly uniform link distances. This behavior is similar to that available in jViz.Rna and valuable for arranging the nucleotides in a relevant, meaningful or simply aesthetically pleasing manner.

### 2.3 Pseudoknots and custom links (Supplementary Material Sections S1.1 and S1.6)

It is often necessary to display the interaction between two molecules or between different parts of the same molecule (i.e. pseudoknots). Although the user can enter pseudoknotted structures in dot-bracket notation (i.e. ((..[[..))..]]), the pseudoknotted nucleotides in these cases are added as links with no strength. One may also add custom links by holding down shift and dragging from one nucleotide to another. This creates a spring-loaded link which can bring distal portions of a molecule together, or connect separate molecules. Such links are useful in depicting RNA–RNA interactions.

### 2.4 Coloring (Supplementary Material Section S1.4)

The coloring of nucleotides is essential for overlaying metadata on top of a structure. forna provides three default coloring modes: position, structure and sequence which color nucleotides according to their position in the molecule, the type of structural element they are in (i.e. stem, interior, hairpin, multi or exterior loop) or their identity (A,C,G or U). A custom coloring mode is provided where bespoke values (as from probing data) can be entered in a text field.

### 2.5 Integrated structure prediction (Supplementary Material Section S1.5)

To simplify the process of going from sequence to secondary structure, forna provides a transparent interface to the Vienna RNA Package (Lorenz *et* *al.*, 2011) which automatically calculates the minimum free energy for a particular sequence if no secondary structure is provided. This allows one to paste a sequence in the input field and immediately view its predicted secondary structure, without using additional tools.

### 2.6 Tertiary to secondary structure (Supplementary Material Sections S1.2 and S1.6)

One of the most important and unique features of forna is the automatic display of secondary structure information given a 3D structure as a PDB file. forna automatically extracts base-pair interaction information using MC-Annotate (Gendron *et* *al.*, 2001) and displays the canonical secondary structure, which can be explored, manipulated or colored as described in the previous sections. Multiple chains are displayed as disconnected graphs. Proteins are displayed as larger gray nodes and interactions between different chains are represented as dashed lines (Fig. 1).

### 2.7 Reusable display container (Supplementary Material Section S2)

Researchers can effortlessly share RNA structures online by adding a few lines of javascript to their web page and showing a diagram of the secondary structure embedded as an SVG container attached to any specified element in the DOM tree. This rendering is purely client side and requires no calls to the server.

## 3 Conclusion

We provide an easy to use, accessible, free, open-source web tool for RNA secondary structure visualization that produces beautiful, highly customizable plots. Our tool requires no externally installed software and is useful for both the exploration and dissemination of RNA secondary structure.

## Supplementary Material

Supplementary Data
