# Supplementary material for: Forna (force-directed RNA): Simple and effective online RNA secondary structure diagrams
Source: Bioinformatics. 2015 Jun 22;31(20):3377–9. doi: 10.1093/bioinformatics/btv372 (PMC4595900; doi:10.1093/bioinformatics/btv372)
Supplement: Supplementary Data [file supp_31_20_3377__index.html]

Forna (force-directed RNA): Simple and effective online RNA secondary structure diagrams — Forna (force-directed RNA): Simple and effective online RNA secondary structure diagrams — Supplementary Data 

# Forna (force-directed RNA): Simple and effective online RNA secondary structure diagrams

## Supplementary Data

files

- Supplementary Data - pdf file
