# Supplementary material for: Forna (force-directed RNA): Simple and effective online RNA secondary structure diagrams
Source: Bioinformatics. 2015 Jun 22;31(20):3377–9. doi: 10.1093/bioinformatics/btv372 (PMC4595900; doi:10.1093/bioinformatics/btv372)
Supplement: Supplementary Data [file supp_btv372_supplement.pdf]

1 Supplementary Material (Gallery)

The implementation and description of `forna` is divided into two parts: the interactive web application (Section 1.1) described in the main text as well as an additional section describing its use as reusable container for displaying RNA secondary structures on web pages (Section 1.2).

1.1 `forna` as a Web Application

1.1.1 Drawing a Secondary Structure Starting from an Open Configuration (Figure 1)

One of `forna`'s key features is the ability to intuitively edit an RNA structure. Links between unpaired nucleotides are added by holding the shift key and dragging from one unpaired nucleotide to another. The structure is immediately updated along with the coloring. The recalculation is performed on the client ensuring lag-free updates. Unwanted links can be removed by holding 'shift' and clicking on them. If the user introduces a pseudoknot with new link, it is detected and added as a force-less link.

```
>molecule_name
CGCUUCAUAUAAUCCUAAUGAUAUGGUUUGGGAGUUUCUACCAAGAGCCUUAAACUCUUGAUUAUGAAGUG
.......................................................................
```

1.1.2 Visualizing a PDB File

PDB files store information about the 3D positions of each atom in a molecule as determined by structural biology methods such as X-Ray crystallography or NMR. While packed with information, they can be difficult to interpret without in-depth knowledge of the structure in question. Extracting the secondary structure requires the use of intermediate programs such as MC-Annotate 2. More recently, `rnapdbee` 1 has been developed as a web service to extract and display secondary structure from PDB files. The resulting images, however, are static and wedded to the layout provided by the visualization tool as well as the secondary structure present in the PDB file.

`forna` extends this functionality by allowing users to input a PDB file and displaying an interactive representation that can be explored and manipulated. Furthermore, `forna` includes information about protein interactions (an interaction in this case denoting the presence of a nucleotide and an amino acid within 2.8 Å of each other). Figure 2 displays the visualization of a Bacterial Ribonuclease P Holoenzyme in Complex with a tRNA. Immediately evident are the interactions between the ribozyme and the 5' and 3' ends of the tRNA as well as the $T\Psi C$ loop. A protein is seen interacting with the large junction and one of the interior loops of the ribozyme.

It should be noted that due to computational constraints, we set a limit of 2MB for the maximum size of a PDB file that can be uploaded. Users wishing to

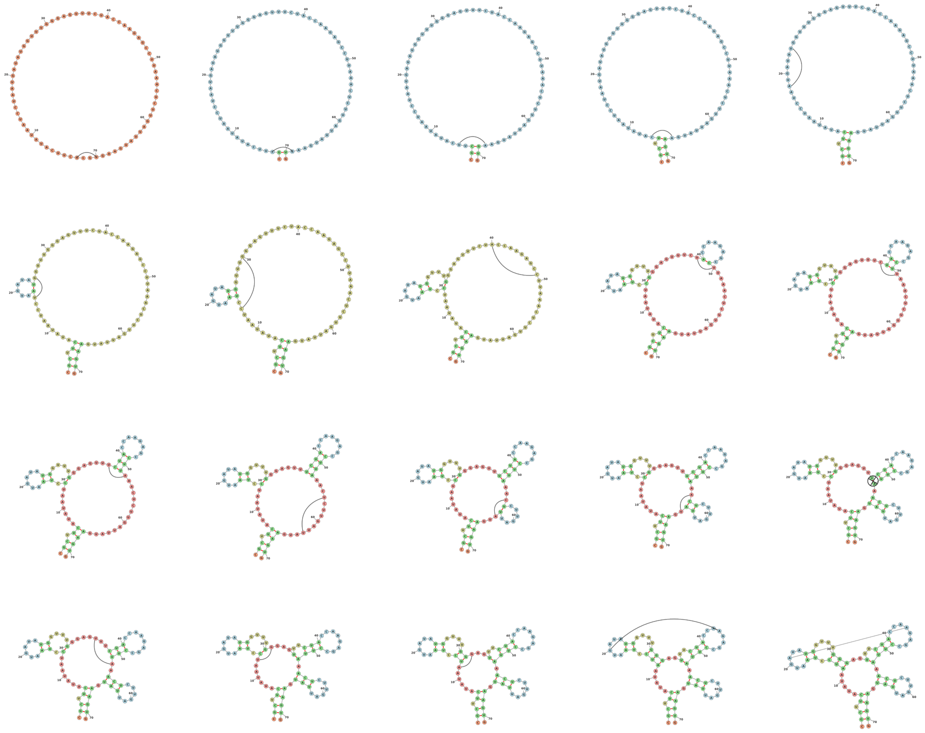

Figure 1: Drawing an RNA from an open configuration using `forna`. This sequence illustrates how one can draw a particular secondary structure and goes from left to right, top to bottom. Each arc represents the newly added base-pair, which is added by shift-clicking on an unpaired nucleotide and dragging to a target nucleotide. In the last column of the third row, a link is removed by holding shift and clicking on the link. In the final step, a pseudoknotted interaction is detected and the resulting link is force-less.

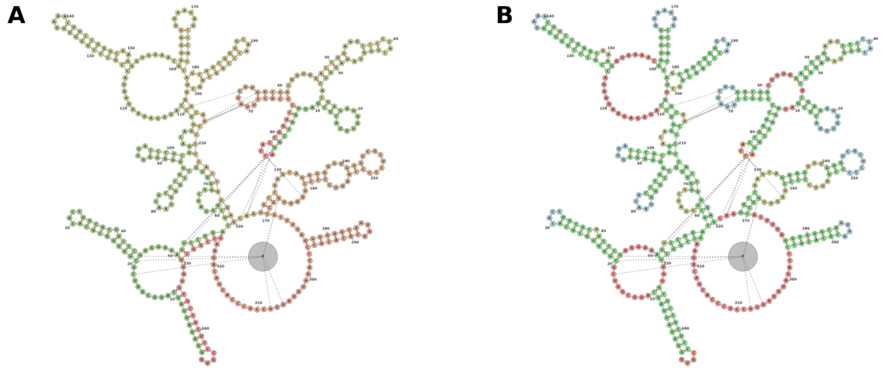

Figure 2: The secondary structure of a Bacterial Ribonuclease P Holoen zyme in Complex with a tRNA (PDB ID: 3Q1Q). The tRNA is shown in the upper right hand corner of each figure while a protein can be found in the middle of the large multi-loop junction. Two coloring schemes are shown, highlighting the positions of each nucleotide within the structure (A) and the type of structure at each nucleotide (B).

visualize larger molecules are encouraged to download and run the forna server locally.

1.1.3 Probing Data

Overlaying chemical probing data on a secondary structure gives researchers an informative perspective of where highly reactive regions lie. In the examples in Figure 3, it is clear that the probing data is consistent with the given secondary structure insofar as the highly reactive regions are unpaired whereas the paired regions exhibit lower reactivity. The example serves to showcase the ease with which probing data can be overlayed onto a given structure. The input sequence and structure for the molecule on the left of Figure 3 are as follows:

```
>GLYCFN_KNK_0002.rdat
ggaaauaaUCGGAUGAAGAUAUGAGGAGAGAUUUCAUUUUAAUGAAACACCGAAGAAGUAAAUCUUUCAGG
UAAAAAGGACUCAUAUUGGACGAACCUCUGGAGAGCUUAUCUAAGAGAUAACACCGAAGGAGCAAAGCUAA
UUUUAGCCUAAACUCUCAGGUAAAAGGACGGAGaaaacaaaacaaagaaacaacaacaacaac
........(((......((((((((......((((((....)))))).(((....(((.....)))...))
)........))))))))...)))..(((((......(((((.....))))).(((....(((....((((.
...)))).....)))...))).......))))).............................
```

The probing data was added by clicking on the 'Colors' drop-up and then on the 'Set' button. The following values (obtained from the RNA Mapping Database `http://rmdb.stanford.edu/repository/detail/GLYCFN_KNK_0002`) were pasted into the field.

```
247.6424 96.2278 54.8271 46.8534 64.6265 21.8767 39.5119 43.1716 14.4877
```

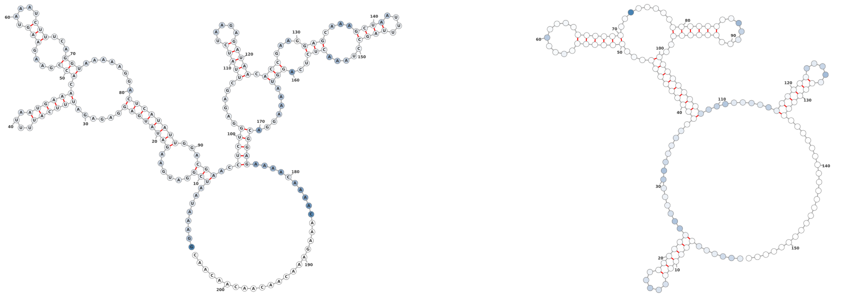

Figure 3: Probing data overlayed onto the secondary structure of an adenine riboswitch (left) and a glycine riboswitch (right). Darker colors indicate higher reactivity.

8.8179 2.8988 3.7053 23.1721 5.0993 3.4704 49.9487 37.8422 6.636 0.6161
0.3902 13.7014 2.9549 -0.376 1.0762 -0.5679 -0.5 2.0993 0.0671 2.4959
12.2261 13.3004 1.4647 -0.4664 -0.9908 0.0252 0.8216 0.7187 2.399 4.2495
4.4933 33.2972 11.1846 1.4183 -1.4358 1.7068 2.6488 0.4078 0.8826 4.6223
-0.0611 -0.8845 1.3948 15.4353 50.3329 6.4028 4.5445 6.3482 0.467 2.1263
39.3715 47.8274 50.979 9.8241 4.3092 0.4139 0.1997 -1.099 4.1683 32.5064
2.5253 -0.4245 10.2521 37.5462 25.9211 30.8512 21.1141 10.5151 -0.4247
1.2889 67.9884 6.7804 -0.2508 -0.1662 2.5511 -0.1782 1.4556 0.6963 -1.0435
-0.3248 4.0919 33.8002 3.647 -0.0516 33.2451 56.4639 8.8362 1.1629 -0.5062
1.1171 -0.2665 -0.5553 0.942 7.709 0.6988 17.9013 6.2786 3.2409 -1.07
0.1746 7.1433 0.1149 2.2959 9.1101 56.9181 56.5 30.1173 27.094 2.0416
6.6394 -0.1116 13.582 8.9534 3.2413 15.9977 0.9295 0.0206 0.7913 50.923
77.8383 -18.9486 7.2471 3.2602 10.3813 1.1856 61.4109 139.4171 131.8496
8.2972 2.6437 3.0427 74.7002 78.0617 5.4987 2.004 1.2521 -1.2755 12.413
0.4201 -0.4107 3.8533 2.0068 71.2049 108.6537 132.3693 6.8693 0.3981
0.0862 -0.773 11.2572 168.3761 -15.3035 14.3589 29.7187 59.0558 101.8314
110.0697 71.0021 1.9045 3.9161 156.4062 4.5086 0.7884 5.374 30.8158
15.1988 151.4786 126.5493 132.0433 150.3512 19.0894 130.44 174.8229
173.997 200.6308 228.1592

On the right of Figure 3 the input structure is:

```
GGAAAGCAAUUCGAGUAGAAUUGGAAAGGGAAAGAAACGCUUCAUAUAAUCCUAAUGAUAUGGUUU
GGGAGUUUCUACCAAGAGCCUUAAACUCUUGAUUAUGAAGUGAAAACAAAGUUAAGGAGUACUUAA
CACAAAGAAACAACAACAACAAC
......((((((.....))))))..............(((((((((...((((((.........))
)))).......((((((.......))))))..)))))))))........((((((.....)))))
)......................
```

The color information is also obtained from the RNA Mapping Database (`http://rmdb.stanford.edu/site_media/rdat_files/ADDRSW_1M7_0006`):

0.2731	0.5547	0.5066	0.2429	0.2110	0.2948
0.0237	0.0312	0.0589	0.0252	0.0250	0.0468
0.4216	0.4143	0.6900	0.4320	0.1618	0.0377
0.0555	0.0813	0.0714	0.0446	0.1151	0.8637
0.8640	0.4475	0.1755	0.1821	0.2529	0.7196
0.8994	0.5176	0.2022	0.2314	0.3745	0.2439
0.0354	0.0274	0.0218	0.0216	0.0222	0.0062
0.0148	0.0195	0.0207	0.0289	0.0232	0.0671
0.0316	0.0206	0.0094	0.0309	0.0296	0.0537
0.0360	0.0419	0.1660	0.0566	0.0546	0.7821
0.0637	0.0417	0.0963	0.0317	0.0280	0.0119
0.0262	0.0161	0.0189	0.0304	0.0490	0.0483
1.8979	0.0314	0.0071	0.0332	0.0305	0.0140
0.0191	0.0173	0.0070	0.0188	0.0159	0.0212
0.0211	0.0793	1.0460	0.6202	0.2630	0.0473
0.0220	0.0267	0.0072	0.0206	0.0175	0.0119
0.0504	0.0431	0.0422	0.0572	0.0379	0.0158
0.0500	0.0235	0.0293	0.0577	0.0413	0.4775
0.5773	0.6706	0.8049	0.2942	0.2483	0.3983
0.2593	0.6670	0.1990	0.0585	0.0596	0.0573
0.0267	0.1003	0.6188	0.2840	0.5888	0.9753
0.1816	0.0147	0.0138	0.0146	0.0152	0.0262
-0.0042	-0.0073	0.0000			

1.1.4 Arbitrary Coloring

It is often useful to color certain nucleotides a particular color to illustrate a region of interest. Figure 4 demonstrates how one can supply coloring information for specific ranges of nucleotides. The secondary structure in this example is extracted from the tertiary structure of the Ternary S-Domain Complex of Human Signal Recognition Particle (PDB ID). The coloring is entered by clicking on the 'Colors' drop-up, clicking 'Set' and then pasting the following text:

```
18-57:red 64-110:blue
```

1.1.5 Kissing Hairpins

One often needs to depict the interaction between two molecules. Figure 5 shows two small molecules interacting via a kissing hairpin interaction. It should be noted that this is difficult to display when the interactions are longer than a few nucleotides due to the layout constraints. Nevertheless, for shorter interactions, adding artificial links can provide an adequate view of where molecules interact. For this example, the following fasta sequences were entered in the 'Add Molecule' dialog:

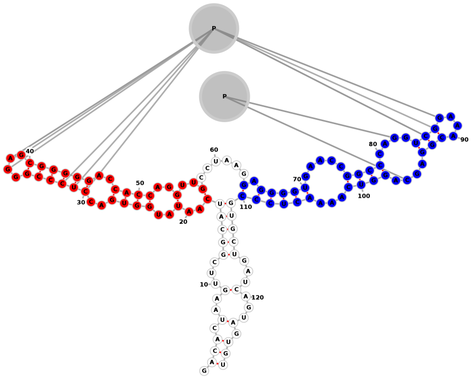

Figure 4: Specifying an arbitrary coloring scheme for an RNA. In this case, the secondary structure of the Ternary S-Domain Complex of the Human Signal Recognition Particle (PDB ID: 1MFQ) is colored to show the two branches which are involved in protein interactions.

```
>a
UCAAAUGAGCUACUCACGUAGCUCAUCCUU
>b
CGAUAUGAGCUACGUGAGUAGCUCAUUGGU
```

The secondary structures are automatically predicted using `RNAfold`. The basepair nearest the hairpin is artificially broken (using 'shift'-click), and extra links are added between nucleotides 13,14,15,16 and 14,15,16,17.

1.1.6 Pseudoknots

Pseudoknots are detected in the input structure using a greedy algorithm which always marks the most nested base pairs as pseudoknots. These nested base pairs are then added as strength-less links and removed from the rest of the structure. Figure 6 shows two examples of structures with pseudoknots, one input as a pdb file (group II intron, PDB ID: 4FAW, left) and the other input from a dotbracket representation (corresponding to an adenine riboswitch). The dotbracket string for generating the structure on the right is shown below. Note the two different types of brackets used ('()' and '[]') in order to denote nested nucleotides. Other brackets such as '{}' and '<>' can also be used to denote multiply nested pairs.

```
>molecule_name
CGCUUCAUAUAAUCCUAAUGAUAUGGUUUGGGAGUUUCUACCAAGAGCCUUAAACUCUUGAUUAUGAAGUG
((((((((((..((((((...[[[...))))))......).((((((..]]]..))))))..)))))))))
```

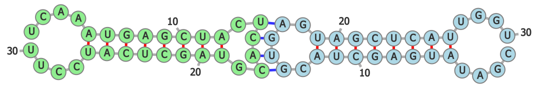

Figure 5: Two molecules interacting via a kissing-hairpin interactions. The inter-molecular base pairs are colored blue, whereas the the intramolecular base pairs are colored red.

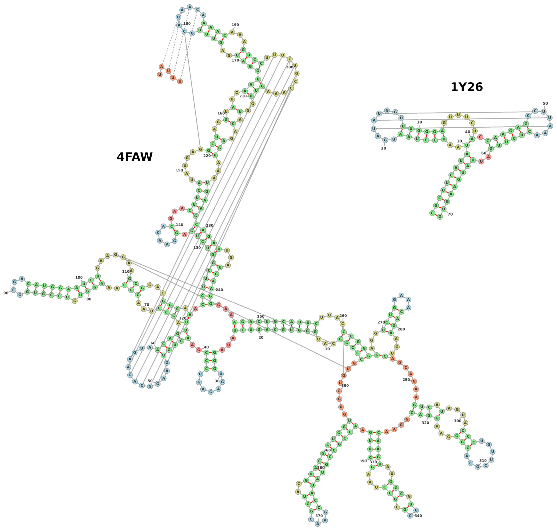

Figure 6: Pseudoknotted structures from an input dotbracket file (1Y26, upper right) and a PDB file (PDB ID: 4FAW, left).

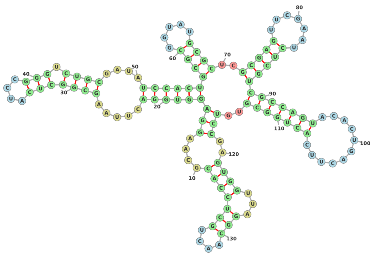

Figure 7: A circular RNA with no external loops and/or 5' and 3' ends.

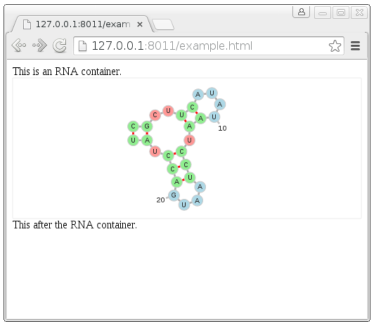

Figure 8: An example of using the `FornaContainer` to easily display an RNA structure within a web page.

1.1.7 Circular RNA

RNA usually exists as a single strand with distinct 5' and 3' ends, but it can also be found as a circular molecule. Such molecules have been ligated at their 5' and 3' ends and thus have no external loops. These can be displayed using forna (Figure 7) by appending an asterisk to the end of the dot-bracket string.

```
>circular_rna
CUGCUCCACGCAAGGAGGUGGACUUAAGCGGCUCAUCCGGGUCUGCGAUAUCCACUGCGCGG
UAUGCGCUCGCGAGUUCGAAUCUCGUCGCCAGUACACUGACUUCACUGGCGUGUCCGAGUGG
UUAGGCAA
..(((((((....(((((((((.....(((((((....))).))))....))))))((((..
...))))..(((((......)))))((((((...........)))))))..)))..))))
...)))..*
```

1.2 `forna` as a Javascript Viewing Container

The web-application version of `forna` described in the main text relies on a server to calculate an initial layout which is then refined by the force-directed layout calculation. It provides an interface for adding and removing structures

as well as for changing the coloring and the display parameters. There are, however, other applications where one may simply want to display a structure without allowing the user to display their own or to change coloring. This is often the case when one wants to share structures online, as for example, from a secondary structure prediction server. To accommodate this need, we provide an independent javascript container which is completely decoupled from the back-end server. The initial calculated layout is simpler, and features such as displaying a PDB file (which require server-side annotation) are disabled, but other features such as panning, zooming and dragging can be enabled using specific parameters.

The container is available as its own repository (called `fornac`: for `fornat` container), and can be instantiated using only a few lines of javascript code. While the specifics of the API are detailed in the online documentation at `https://github.com/pkerpedjiev/fornac`, The general pattern for use is shown in the example web page below:

```
<!DOCTYPE html>
<meta charset="utf-8">

This is an RNA container.
<div id='rna_ss'> </div>
This after the RNA container.

    <link rel='stylesheet' type='text/css' href='css/fornac.css' />
    <script type='text/javascript' src='js/jquery.js'></script>
    <script type='text/javascript' src='js/d3.js'></script>
    <script type='text/javascript' src='js/fornac.js'></script>

    <script type='text/javascript'>
        var container = new FornaContainer("#rna_ss",
            {'applyForce': false, 'allowPanningAndZooming': true});

        var options = {'structure': '((..((....)).(((....))).))',
                        'sequence': 'CGCUUCAUAUAAUCCUAAUGACCUAU'
        };

        container.addRNA(options.structure, options);
    </script>
```

The two key features of the example are the `div` to contain the forna container and the javascript at the bottom which populates it with an RNA sequence, secondary structure and some optional parameters. The resulting web page can be seen in Figure 8 where a visualization of the RNA secondary structure appears without the need to first create a static image or call a java library.

References

[1] M. Antczak, T. Zok, M. Popenda, P. Lukasiak, R. W. Adamiak, J. Blazewicz, and M. Szachniuk. RNApdbee - a webserver to derive secondary structures from pdb files of knotted and unknotted RNAs. *Nucleic acids research*, page gku330, 2014.

[2] P. Gendron, S. Lemieux, and F. Major. Quantitative analysis of nucleic acid three-dimensional structures. *Journal of molecular biology*, 308(5):919–936, 2001.
